# Factors influencing publication choice: why faculty choose open access

**DOI:** 10.1186/1742-5581-4-1

**Published:** 2007-03-09

**Authors:** Stefanie E Warlick, KTL Vaughan

**Affiliations:** 1 Liaison & Outreach Services Librarian, Health Sciences & Human Services Library, University of Maryland, Baltimore, Maryland, USA; 2 Librarian for Bioinformatics & Pharmacy, Health Sciences Library, UNC-Chapel Hill, USA

## Abstract

**Background:**

In an attempt to identify motivating factors involved in decisions to publish in open access and open archives (OA) journals, individual interviews with biomedical faculty members at the University of North Carolina at Chapel Hill (UNC-Chapel Hill) and Duke University, two major research universities, were conducted. The interviews focused on faculty identified as early adopters of OA/free full-text publishing.

**Methods:**

Searches conducted in PubMed and PubMed Central identified faculty from the two institutions who have published works in OA/free full-text journals. The searches targeted authors with multiple OA citations during a specified 18 month period. Semi-structured interviews were conducted with the most prolific OA authors at each university. Individual interviews attempted to determine whether the authors were aware they published in OA journals, why they chose to publish in OA journals, what factors influenced their publishing decisions, and their general attitude towards OA publishing models.

**Results & Discussion:**

Fourteen interviews were granted and completed. Respondents included a fairly even mix of Assistant, Associate and Full professors. Results indicate that when targeting biomedical faculty at UNC-Chapel Hill and Duke, speed of publication and copyright retention are unlikely motivating factors or incentives for the promotion of OA publishing. In addition, author fees required by some open access journals are unlikely barriers or disincentives.

**Conclusion:**

It appears that publication quality is of utmost importance when choosing publication venues in general, while free access and visibility are specifically noted incentives for selection of OA journals. Therefore, free public availability and increased exposure may not be strong enough incentives for authors to choose open access over more traditional and respected subscription based publications, unless the quality issue is also addressed.

## Background

In recent years, open access and open archives (OA) publishing has become a 'hot topic' for university librarians, faculty, and administrators. In particular, open access publishing has become increasingly popular within the biomedical sciences. From 2003 to 2005 approximately 22% of articles written by faculty at the University of North Carolina at Chapel Hill (UNC-Chapel Hill) and indexed in PubMed MEDLINE were published in "free full text" journals.

There is disagreement as to what constitutes an "open access" journal. The most liberal definitions allow for free full text access, possibly after a relatively brief embargo period. Other definitions require immediate free publication. The most stringent definitions require both immediate free publication and copyright retention by the author(s). For example, the Budapest Open Access Initiative (BOAI) [1] defines open access as:

"Free availability on the public internet, permitting any users to read, download, copy, distribute, print, search, or link to the full texts of these articles...without financial, legal, or technical barriers other than those inseparable from gaining access to the internet itself. The only constraint on reproduction and distribution, and the only role for copyright in this domain, should be to give authors control over the integrity of their work and the right to be properly acknowledged and cited."

Regardless of definition, it is clear that open access publishing is in stark contrast to the traditional publishing model in two possible ways.

1) Access to articles published within these journals is free of charge to the public readership.

2) Copyright restrictions on authors may be removed, and authors retain rights rather than automatically transferring them to publishers.

Although the benefit OA publications provide to the world of scholarly communication in terms of accessibility and information dissemination may be obvious to proponents, barriers such as "significant reservations about quality and preservation" have left many authors unconvinced [2]. In order to encourage publication in open access journals and diffuse "concerns or objections" pertaining to the peer-review quality of OA publications, initiatives such as the Public Library of Science (PLoS) [3] and BioMed Central (BMC) [4] have been launched in recent years [5]. In addition to larger scale initiatives such as PLoS, many universities have created programs which provide guidance and support to authors exploring publication in open access journals. For instance, the North Carolina State University Libraries Scholarly Communication Center [6] "provides workshops and presentations on copyright, fair use, and other scholarly communication topics," as well as individual consulting for faculty. Furthermore, recent research comparing the impact of open access citations against those published in traditional journals has been conducted in order to identify any significant differences. Multiple studies have reported that the impact of open access citations often surpasses those published in non-OA publications [7-9]. For example, in 2004 Antelman [7] concluded that "across a variety of disciplines, open-access articles have a greater research impact than articles that are not freely available."

While extensive literature can be found focusing on the impact of open access publishing from the point of view of libraries and publishers (often focusing on financial factors), until recently there was little that studied the authors who have chosen to publish their works in these publications [10]. Identifying factors involved in author decisions to publish in open access journals helps illuminate issues that may encourage or discourage author support of open access publishing models. Further understanding of these issues can assist the efforts to improve author perceptions of and confidence in open access publications.

### Literature Review

In addition to discussions about how to define open access, much of the literature about OA publishing focuses on the relative importance of specific issues such as impact factor, publication speed, and author fees [11]. Increasingly the body of research focused on author attitudes towards these issues is expanding. Some have argued that actively publishing researchers are at the heart of the open access movement and it is important to "consider the wants and needs of authors" [12].

Fueling the recent shift in focus to author publishing behaviors and attitudes are several large scale studies which report on the experience and opinions of authors and open access publishing [2,13]. Findings suggest that although deliberate open access publishing continues as a minority activity amongst publishing authors, there has been a fairly significant rise in non-OA author awareness of open access and related issues [2]. Of particular interest are the issues reported as most meaningful and significant to authors when making decisions on where to publish their work.

In an extensive 2004 survey [5] comparing the experience of approximately 100 OA authors and the same number of non-OA authors, Swan and Brown presented findings on reasons authors choose open access venues for publication. Free access (92%), speed (87%), and wide-audience (71%) were reported as most important. More recently Rowlands, Nicolas, and Huntingdon, found prestige of the publication based on reputation or impact factor, as well as type of research and speed, to be essential in the decision making process for all authors [2].

### Free Access

Free access for readers undoubtedly serves as an enticing point in the promotion of open access publications. In a 2004 study, three-quarters of the surveyed authors reported free access as the "strongest characteristic associated with open access journals" [10]. Similarly, in 2004 it was found that 90% of the authors surveyed acknowledged having chosen to publish in open access journals on the basis of free access [13].

Individual proponents of the open access movement have argued that open access grants accessibility to institutions and individuals with limited resources, as well as encourages the sharing of original research on an international level [14,15]. Lawrence Lessig, a vocal proponent of the open access movement, argues that the real objective of open access is not to undermine traditional publishers, but rather aid in the distribution of a work "as widely as possible around the world" [16].

### Publication Quality

Open access publications continue to face criticism regarding quality and prestige when compared to more traditional and established journals [10,12]. Author perception of publication quality appears to be based on various factors including the peer review process and the reported impact factor of a journal.

The JISC/OSI report found that while many non-OA and OA authors are aware of the perception that the peer-review quality of open access is lower, the OA author responses supported previous reports that the peer review process within open access journals has been quite similar to that of traditional publications [5]. In addition, it seems that most authors continue to stress the importance of retaining peer review in order to maintain the quality of all publications [10].

The impact of individual publications has and continues to be a heavily relied upon indicator of journal quality. Whether based on a perception of or on the official Impact Factor, it is important to many authors and often attributed to career success to publish in high impact journals [13]. As with perceived peer-review quality, impact factor has been cited as a disincentive in regards to open access publishing [13]. Individual impact or the quantity of citations per individual work is also of interest to authors. However, recent studies have shown that articles in open access journals may have increased visibility and in turn a higher rate of citation compared to non-OA peer articles [7,17,13].

### Speed

The amount of time between acceptance of an article and its publication is often listed as important in the consideration process. This is particularly true for those in more competitive research areas such as the sciences [18]. OA authors have reported that in their experience, open access publications are "faster" compared to traditional publications [13].

### Cost

One controversial outcome of the open access movement has been a heavy reliance on an author pays model. Within this business model, authors are charged per accepted publication in order to subsidize journal costs traditionally supported by subscription fees. Evidence has shown that many authors are in opposition to these fees and have reported that they are not prepared to pay for open access publishing [10]. Some have vocalized concern for the ability of authors to cover these costs, while others argue that it is a matter of author willingness to reallocate research funds [19,20]. Nicholas & Rowlands note that the author pays model may be misinterpreted by some who are not aware that most funding sources will cover publication costs such as page charges and author fees [10].

The significance of author fees as a discouraging factor may be influenced by complex factors including the author's field, tenure, and availability of external funding such as grants. In a comment based on recent reports on the effects of open access publishing, a UNC-Chapel Hill tenured faculty member in the biological sciences observed that "for the price of a set of old-fashioned reprints, an author can make an article open access, be virtually assured of a larger readership, and have a high probability of increased citations levels" [20].

### Copyright

At the 2005 UNC-Chapel Hill Scholarly Communications Convocation [21], the director of the UNC-Chapel Hill Law Library, Laura Gassaway, argued that benefits of faculty copyright retention extend to "individual authors, other faculty, the institution and the research community" [22].

While explicitly required in the BOAI [1] definition of open access, copyright retention does not consistently appear as a motivating factor for OA publishing. In a 1999 survey of authors, Swan reported that although more individuals were interested in retaining the copyright on their works, this feeling was less prevalent in the sciences than the arts [18]. Another more recent survey showed that in general authors do not value the opportunity to retain copyright or request reproduction permission from publishers [2].

## Methods

This exploratory research study consisted of individually conducted semi-structured interviews with biomedical faculty at Duke University and UNC-Chapel Hill, two large research institutions. The seventeen interview questions used stemmed from four original questions: Is the author aware that she/he has published in open access/archives journals; If so, why did he/she chose to publish in an open access journal; What factors influence their publishing decisions; and, What is their general attitude towards open access publishing models. Approximately half of the questions solicited open-ended responses. The questions were reviewed and revised by the UNC Health Sciences Library Scholarly Communication Committee and approved by Internal Review Boards (IRB) at both institutions.

Potential subjects were recruited from a gathered list of biomedical faculty at UNC-Chapel Hill and Duke. Faculty members with multiple publications in true open access (free from date of publication) or open archive (free after an embargo period) journals within the eighteen month period January 2004 to June 2005 were included. These publications were identified using a search of the PubMed Medline database [23] for "free full text [sb] AND chapel hill [ad]", using UNC as an example, along with a supplemental search of the PubMed Central database [24] to capture any additional authors at UNC-Chapel Hill and Duke. Citations from PubMed and PubMed Central were used in order to identify authors having explicitly noted affiliation with the two institutions. The majority of the recruited participants had four or more open access or open archive publications during the time period.

Starting with the most prolific of the identified authors, e-mail recruitment letters were sent to 35 UNC and 22 Duke faculty. Each of the interviews was conducted or collected by the principal investigator and lasted approximately thirty minutes. If a participant was unavailable for an interview, she/he was asked to complete the electronic set of questions and return them via email.

Prior to each interview, the principal investigator collected specific publicly available information about each participant, including name of affiliated department, faculty status, PubMed citation list of all (both OA and non-OA) publications during the 18 month period.

ATLAS.ti, a qualitative analysis program for coding and interpreting text, was used for coding and analysis of interview data. All responses to open ended questions were tagged with identified key concepts. When responses did not fit into existing categories, new concepts were created. Many of the concepts such as quality, speed, impact factor, and cost were topics related to open access publishing identified in previous reports [5,25].

## Results & Discussion

Of the fifty-seven biomedical faculty recruited for this study, fourteen interviews were granted and completed. Eleven of the participants were from UNC-Chapel Hill and three from Duke. Six (43%) of the interviews were conducted in-person, two (14%) over the phone, and six (43%) were filled out by the participant and returned via email. Four interview participants were assistant professors, four were associate professors, and six were full professors. Affiliated departments of the faculty participants included Medicine (7), Biology (4), and Public Health (3). The primary communicated response for declined participation was lack of free time to participate.

Each of the participants were presented with seventeen interview questions and asked to elaborate as much as appropriate for each response.

### Q1: How do you decide where to submit your articles for publication? (see Table 1)

**Table 1:** Deciding factors in order of frequency

Impact Factor	7
Target audience	6
Prestige	4
Topic	3
Cost	2
Speed of publication	2
Quality	2
Visibility	2
Open Access status	2

Additional participant comments regarding publishing decisions:

"Audience I want to meet, first by a wide margin."

Authors discussed the factors important to their personal decision making process. Some respondents simply listed factors while others ranked the factors in order of importance. It appears that when making decisions on where to publish, authors rely heavily on their own perception of specific factors.

Impact factor and audience were mentioned most frequently across participants. Many of the factors listed above can be sorted into some common categories. For example, the overall quality of a publication relates to the perceived impact factor as well as level of prestige and general quality. Target audience, topic of the published work, and visibility refer to publication readership. In addition, there are logistical issues such as author fees and the speed of the publication which affect publishing decisions.

### Q2: Does speed of publication influence your choice of journal? (see Table 2)

**Table 2:** Speed important?

Yes	13
No	1
n = 14	

Additional participant comments regarding speed:

"Now it doesn't feel like there is a huge difference in speed because most have gone to electronic review and submission."

"Speed used to be more of a factor during my post-doc."

"Everyone tries to avoid the journals known to be slow or disorganized."

"It is more the speed of review and notification that is important to me."

"The advent of electronic reviewing has sped up and homogenized the process in my field."

As mentioned, the speed of a publication appears to be an important factor for active authors, especially those in competitive fields of research. An explanation of "speed" was not provided to participants. Responses indicate that speed is often related to the submission, notification, and peer-review processes.

The majority of authors reported that speed is in fact important when selecting journals for publication submission. From participant responses to this question, it appears that in the past there was significant variation in speed across publications. The online environment, regardless of "openness" of publication, seems to help alleviate some of the problems that occurred in recent years.

### Q3: How important is impact factor? (see Table 3)

**Table 3:** Impact factor

Quite important	8
Moderately important	3
Not a factor	3
n = 14	

When considering the importance of impact factor, one respondent acknowledged that it can be used as a crutch to help weed through the increasing amount of literature that is published. Another argued that the impact factor of a publication correlates with the level of exposure an article will receive.

Reponses to Q1 and Q3 indicate that impact factor is often a point of consideration in publishing decisions for biomedical faculty at both UNC-Chapel Hill and Duke. If impact factor continues to strongly influence author perception of journals, open access publications will have to establish credibility through this measure and remain competitive with the more traditional high impact publications.

### Q4: Are you influenced by the number of subscribers/readers that a journal reports? (see Table 4)

**Table 4:** Influenced by reported subscribers?

Yes	3
No	11
n = 14	

Many of the respondents acknowledged that they did not know how to go about finding this kind of number or report. Another acknowledged that this may be a more generational factor and that the newer generation of faculty may not be as interested.

Overall, the number of subscribers and readers reported by a journal does not seem to sway decisions on where to publish. The impact factor of a journal appears more influential to these authors.

### Q5: What is your general attitude towards open access publishing models?

For this question, participants were provided with a brief description of how open access has been defined within this study. Those who filled out questions via email were provided with a definition along with the set of questions. Twelve of the fourteen participants specifically noted that they were in favor of the open access approach to scholarly publishing. Respondents cited increased visibility, information dissemination, and free access as positive aspects of open access. While some of the participants revealed that a positive attitude towards open access does not actually influence their publishing decisions, awareness of relevant issues and an articulated favor towards open access models is promising.

Additional participant comments regarding their attitude towards open access:

"It will increase visibility of research findings."

"I think they're good if the journal is good, but it largely doesn't influence me one way or another."

"I think of it as a highly beneficial development with wide-ranging impact on the practice of science."

"It is the best way to disseminate scientific information in an equitable manner. Not everyone can afford site licenses or subscriptions that are required if you don't have open access."

"If I had a choice between publishing in an open access or a non-open access journal that were roughly equivalent, I would choose open access."

"I publish all my articles in open access when I can."

"I think things should be open, but I also understand that journals have to survive. I think the policy of a 6 month embargo is fine, but it is mostly an irrelevant issue at UNC because they virtually have open access to everything through the library."

### Q6: Is publishing in an OA journal an important part of the "where to publish" decision? (see Table 5)

**Table 5:** OA part of publishing decision?

Yes	9
Increasingly	2
No	3
n = 14	

Additional participant comments regarding open access as a part of publishing decisions:

"Increasingly important because things are moving more quickly now that they are online."

"I see it being more of an issue in the future."

While all participants were previously identified as OA authors, it was unknown whether each had made a conscious decision to publish in an open access journal. A majority of the UNC-Chapel Hill authors and all of the Duke authors reported that at this time the open access status of a journal does influence publishing decisions. Two participants noted that open access had not been much of an influential factor in the past, but has recently become more important to them as authors. Based on comments from these two authors, it seems an increased awareness of open access as a publishing consideration may be a result of the evolving online publishing environment.

### Q7: If so, what is your motivation for publishing in an OA venue? (see Table 6)

**Table 6:** Motivating factors of OA in order of frequency

Free Access	9
Visibility	3
Speed	2
Antipathy towards traditional publishers	2
When targeting a general (non-professional) audience	1

Additional participant comments regarding personal motivation for open access publishing:

"Ensure wider access of results that are worth publishing and to modernize the way information is shared on a global scale."

"In theory, data is disseminated more easily because anyone can get it."

"Once you get past the most elite journals in the field, there is almost no reason not to publish in open access."

Of the participants answering "yes" or "increasingly" to Q6, the five factors were listed as reasons for considering open access when making decisions about where to publish.

Similar to other reports on author attitudes towards OA publishing, a majority of the UNC-Chapel Hill authors (55%) and all of the Duke authors identified free access as a motivation associated with open access. Speed and visibility were mentioned by a minority of participants.

### Q8: Are there any incentives for you to publish in an open access venue? (see Table 7)

**Table 7:** Open access incentives in order of frequency

Audience accessibility	7
Broad exposure	5
Not that I know of	4
Retain copyright	3
Rapid dissemination	2
UNC covers author fees	1
Reviewer comments posted	1
High quality publications	1

Additional participant comments regarding open access incentives:

"Open access is practical in terms of audience and accessibility and philosophical in terms of ownership."

"Retaining copyright makes life easier."

"Anyone can read it from anywhere, including me!"

In an attempt to identify possible points for open access promotion, participants were asked to list any incentives for OA publishing.

Four UNC-Chapel Hill authors could not come up with any incentives to publish in an open access venue. Some of the four mentioned that instead there are disincentives present (see Q9). Audience accessibility, in terms of free and/or easy online access, and broad exposure were most often noted as OA incentives. Three of the responses to Q8 included copyright retention as an incentive of open access publishing. While copyright retention was not reported as a motivating factor for OA publishing (Q7), it is important to note that it is viewed as an incentive for some.

### Q9: What disincentives are there for you to publish in an OA venue? (see Table 8)

**Table 8:** Open access disincentives in order of frequency

Cost	5
Publications not highly respected	4
Few venues	3
Not that I know of	3
Lower impact factor	1
Concerns for own career	1
Concerns for career of their students	1
Lack of OA support from institution	1

Additional participant comments regarding disincentives for open access publishing:

"Page costs may be a factor, but UNC is a member [of the open access publisher] so it doesn't matter. I have to deal with high page costs anyway because I have to pay for color images."

"In some cases, journals increased page charges to offset the costs of open access. This can be a factor as the amount of money we have for page charges is small and comes from grants, not our department or UNC funds."

"Cost. For example PLoS is very expensive, but the University has an agreement so I pay less."

"None, except some open access venues are not highly respected."

Many of the respondents noted that these factors are known perceived disincentives and are not reflective of their personal feelings towards open access. However, some authors reported few venues and lower impact as disincentives directly affecting their own publishing. Four of the five participants who mentioned author fees acknowledged that cost is also an issue in traditional publishing models. While only three of the eleven (28%) UNC-CH participants mentioned author fees in their response to Q9, two of the three (66%) Duke participants noted cost as a disincentive for OA publishing. Two additional participants mentioned that there are no disincentives, specifically since the cost ends up being similar for faculty who use color images or otherwise incur page charges in traditional journals.

### Q10: Does your department make a statement for or against open access publishing? (see Table 9)

**Table 9:** Departmental Statement

Yes	1
Not that I know of	5
No	8
n = 14	

Only one respondent reported that his department has made a statement regarding open access publishing. In this case, the noted departmental statement was pro-open access. Three respondents from the same academic department at UNC-Chapel Hill mentioned that while there is no formal departmental statement, specific individuals within the department are vocal and encourage open access publishing amongst their colleagues.

### Q11: Are you aware that there was an open access convocation on the UNC-Chapel Hill campus? (see Table 10)

**Table 10:** Aware of the UNC-Chapel Hill Convocation?

Yes	1
No	13
n = 14	

In January of 2005 UNC-Chapel Hill hosted a one and a half day long campus-wide Scholarly Communication Convocation. Issues addressed at the convocation included copyright, institutional repositories, and open access publishing. This question attempted to identify whether the faculty participants were aware of and/or participated in the Convocation.

Only one out of the eleven respondents was aware of the 2005 Convocation. The one author who answered "yes" to this question attended the conference and noted that he did not believe that there were many actively publishing authors in attendance. This question was specifically targeted at the UNC-CH participants, but was also presented to Duke participants. All of the Duke participants responded No.

### Q12: Have you published in any open access journals? (see Table 11)

**Table 11:** Published in an open access journal?

Yes	11
Not that I know of	3
n = 14	

Q12 and Q13 were asked in order to determine if the authors made conscious decisions to publish in an open access venue.

The large majority of UNC-Chapel Hill authors and all of the Duke participants were aware of their OA publications. In some responses, authors indicated that they chose a publication specifically based on the OA status. Open archive publications with policies to release items as freely available after a specified amount of time were also included in the recruitment. It is likely that some of the authors were unaware that they had published in an open access or open archive venue because the articles were not freely available at the time of publication.

### Q13: If so, were you responsible for selecting the publication? (see Table 12)

**Table 12:** Selected OA publication?

Yes	13
No response	1
n = 14	

All but one author indicated responsibility for selecting the open access journals where their work had been published. Two of the "not that I know of" respondents from Q12 answered "yes" to this question, indicating that they were responsible for choosing the publication but were unaware of the OA status of the journal.

### Q14: If not, do you know why the decision was made to publish in an OA journal?

There were no responses to this question. All but one participant responded "yes" to Q13, making Q14 irrelevant. The one of the participants who submitted the interview electronically via email, did not respond to this question.

### Q15: Were you or any of the other authors responsible for paying author fees? (see Table 13)

**Table 13:** Paid author fees?

Yes	11
No	2
Can't recall	1
n = 14	

Additional participant comments regarding author fees:

"Yes initially, until UNC assumed that role."

"We have to pay for page charges in most journals anyway...NIH and most other grant sources just get a bulk budget. It is assumed that you will be paying those charges."

As discussed earlier, some open access journals require authors to pay fees for each publication. In addition to finding out whether or not participants were required to cover author fees when publishing in OA journals, Q15 and Q16 attempted to identify whether or not the author fees associated with open access publishing are a concern for UNC-Chapel Hill and Duke University authors.

The majority of UNC-Chapel Hill participants and all of the Duke participants were responsible for paying author fees. Two of the authors acknowledged that for some of their open access publications, author fees were fully or partially alleviated because of UNC-Chapel Hill institutional memberships with BioMed Central and PLoS. Two of the three authors who answered "no" to the question "have you published in any open access journals?" (Q12), answered "yes" to this question (Q15). These responses indicate that author fees are not limited to open access publishing venues.

### Q16: If so, where did the funding come from? (see Table 14)

**Table 14:** Funding Source

Grants	11
N/A	3
n = 14	

All respondents answering "yes" to Q15 reported that author fees were covered by grant funding. It appears that when author charges are required for either open access or more traditional publications, biomedical faculty at UNC-Chapel Hill and Duke University have no trouble covering the costs with grant funding.

### Q17: What else do you have to share about open access and publishing?

This question was an opportunity for participants to provide additional commentary on open access issues. They were not asked to provide solutions or possible next steps for the open access movement, but rather to identify areas of the open access conversation that were not covered by previous interview questions. A few of the most interesting comments are listed below.

Some commentary was consistent across multiple participants. For example, three individuals mentioned that they hope open access will become the "norm" within publishing. While some respondents stated that they think this will happen regardless, others believed OA should be encouraged and championed by progressive faculty. Two suggested the maintenance of a rigorous peer-review process as the key to the success and progression of the open access publishing movement. Along the same lines, open peer review and the inclusion of reviewer comments with article publications was noted as a helpful feature found within some open access publications.

Two participants mentioned that while they encourage and promote open access, they also understand the business side of publishing and the coinciding factors requiring consideration. The open access business model of the Proceedings of the National Academy of Sciences (PNAS), requiring authors to pay $1,000 to make an article freely available from the date of publication, was criticized by one author who believed the six month embargo period was sufficient to satisfy the desire for free access to the public.

A number of participants brought up issues concerning copyright and copyright retention during the interviews. For example, copyright retention does not appear to be a high priority for publishing authors. Instead, author participants openly discussed sharing copyright protected material when they have received requests for copies of their own publications from interested individuals. Such comments match with those from other studies.

An additional point of view presented within Q17 was concern regarding possible consequences for junior faculty publishing in open access venues. As tenure and promotion is a serious subject within academic institutions and open access publishing is not yet a widely accepted activity, it is reasonable to question the effect it will have on new faculty. While this is a legitimate consideration, when asked about open access publishing disincentives (Q9), only one participant mentioned concern for career.

Additional participant comments regarding open access and publishing:

"If it were up to me, I would only publish in open access journals."

"I understand that we have to preserve peer-review and that the key to the success of open access is to keep it paired with peer-review."

"Although this may not be a feature shared by all open access journal, I am appreciative of the identifiable peer reviews. In my experience these have been considerably more thorough and thoughtful than the de-identified reviewer critiques we typically get from "conventional" journals."

"Journals like PNAS confuse me when they try to charge authors $1000 to have it immediately open."

"Embargo does not mean much to me because most of the people that will be reading my work are at institutions that have access to the material. Sometimes people will contact me for copies of the articles, so I end up breaking copyright a lot."

"I send pdfs to those who need access...I receive a reasonable amount of these request, mostly from foreign countries."

"I initially got interested in open access because of requests for pdfs from researchers and students in Eastern Europe and Russia who could not afford subscriptions to journals."

"I have never paid to have my research published, but I also have signed over all copyright to the professional journals...which seems to be the tradition for medical journals."

"Another issue that has been brought up is with junior faculty. Is it a good idea for them to publish in open access journals? It could be dangerous."

## Conclusion

Data analysis of participant responses to interview questions attempted to gain qualitative insight into personal accounts and perceptions of publishing decisions and trends. Knowledge of author attitudes towards open access publishing models will help OA proponents focus on factors that are meaningful to a specific population of authors and avoid futile efforts.

Similar to previous findings, the idea of free access for all appears to be an important motivational factor and incentive for open access publishing. Many of the authors who did not acknowledge open access as a part of the "where to publish decision" noted accessibility as an incentive to publish in OA journals. As reported by some of the participants, requests for electronic copies of their publications by individual researchers, often in foreign countries, remains quite frequent and in some cases has prompted an interest in the open access movement. Affiliates of UNC-Chapel Hill and Duke University benefit from access to an extensive amount of expensive information resources, raising awareness of the information barriers faced by researchers and authors at less fortunate national and international institutions.

In regard to the general quality of publications, multiple authors stressed that open access publications are often considered to be less respected than established journals in their own fields of research. Maintenance of rigorous peer review was suggested as one way to ensure and promote the quality of OA publications. In addition, open peer review and the posting of editorial comments were cited as valuable features available within some open access journals. The impact of open access promotion through individual author advocacy amongst departmental colleagues was also apparent within participant commentary. Encouraging vocal advocates and early adopters to share positive open access publishing experiences, and highlighting OA journals which have successfully maintained stringent peer review may dissolve some of the criticism towards the quality of OA venues.

It is clear that many of the authors place value on either the reported impact factor or the perceived impact (based on either peer or personal opinion) of publications. Also, it appears that, at least among authors at UNC-Chapel Hill, there is a continued belief that open access publications have a lower impact factor than traditional journals. As OA publications are increasingly included in the annual ISI Journal Citation Report, it will be interesting to determine if these presumptions are accurate. A recent report from the Public Library of Science (PLoS) revealed that one of their high profile open access publications, *PLoS Biology*, was found to have a much higher impact factor than many of its peer journals in general biology [26]. As impact is an important publishing factor for many biomedical authors at both UNC-Chapel Hill and Duke University, it will be essential to monitor future reports on the impact factors of OA publications compared to traditional journals, as well as the citation level impact of individual articles appearing in open access publications.

The majority of respondents acknowledged the importance of speed in the publication process. However, in contrast to reports from earlier research, there was no clear indication that speed was specifically correlated with publishing in open access venues [13]. While participants were not asked whether they attribute speed of publication to open access venues, responses indicate that the speed of submission, notification, and review processes have become less of an issue for all publications as the online publishing environment evolves. Therefore, the speed of OA journals as compared to more traditional journals may not be an appropriate point of promotion as an open access publishing incentive. The *PLoS One *[27] model of open peer review, in which an essentially unedited version of a paper is "published" very rapidly, then discussed in an open forum, may further push the idea of speed to publication. In the traditional peer review model, however, it is difficult to imagine how OA publishing may improve on other electronic models.

Although previous findings have indicated an unwillingness to accept author pays models, it appears that for the most part UNC-Chapel Hill biomedical faculty are unconcerned with publication charges associated with either traditional or open access publications. Instead, there seems to be an expectation that some charge will be required for each accepted publication. This finding may not ring true for faculty at Duke, as two out of the three participants noted cost as a disincentive. Both institutions are strongly funded by federal and corporate grants, particularly in biomedical research areas. According to the UNC-Chapel Hill Office of Sponsored Research Annual Report [28], sponsored funding reached nearly $580 million in FY2005, while the Duke University Financial Services reported a total of approximately $697 million in grants, contracts and similar agreements [29].

One difference between the two institutions is that UNC-Chapel Hill covers the cost of publishing in BioMed Central journals whereas Duke does not. It may be that Duke authors are prompted to pay for publishing more often than authors at UNC-Chapel Hill because of this difference in institutional funding support. While cost concerns for open access publishing models should not be discounted for all authors, it may not be an issue for biomedical faculty at major research institutions such as UNC-Chapel Hill and Duke due to the level of funding granted on a yearly basis. In addition, direct and indirect funding support on the part of universities may further allay authors' concerns about page charges. One can imagine a situation where it could be more expensive for an author to publish in a non-OA journal, because of lack of institutional support, than to publish in a page charging OA journal.

Copyright retention is apparently a known but unimpressive incentive for open access within this specific population of authors. UNC-Chapel Hill study participants brought up the issue of copyright on several occasions. Four authors specifically mentioned that they have broken copyright and two more implied that they have done so in the past. Neither copyright retention nor infringement were mentioned by the Duke participants. While it is interesting that some of the authors were conscious of alternative intellectual property models, copyright restrictions seem to have little impact on selection of publishing venues.

When targeting biomedical faculty at UNC-Chapel Hill and Duke, speed of publication and copyright retention are unlikely motivating factors or incentives for the promotion of OA publishing. In addition, author fees required by some open access journals are unlikely barriers or disincentives. It appears that publication quality is of utmost importance when choosing publication venues in general, while free access and visibility are specifically noted incentives for selection of OA journals. Free public availability and increased exposure may not be strong enough incentives for authors to choose open access over more traditional and respected subscription based publications, unless the quality issue is also addressed.

This exploratory research study was limited to a small population of biomedical faculty at two large research institutions. Findings within this report may not be applicable within other populations. The authors would be interested in consulting with researchers at other types of institutions to extend this research beyond these two large research universities. While the majority of participants identified themselves as open access proponents, the inclusion of open archives authors provided some insightful variation in author responses to interview questions. As more citations within true open access publications become available, it will be interesting to limit interview participants to OA authors who have published in journals freely available to anyone from date of publication. Future research focused on non-OA authors may also provide greater understanding of personal open access disincentives such as consequences related to the promotion of junior biomedical faculty.

## Competing interests

The author(s) declare that they have no competing interests.

## Authors' contributions

SEW carried out the individual interviews, performed the analysis and drafted the manuscript. KTLV participated in the design and coordination of the study, served as a consultant during the data analysis and helped to draft the manuscript. Both authors read and approved the final manuscript.

## References

[B1] Budapest Open Access Initiative. http://www.earlham.edu/~peters/fos/boaifaq.htm#openaccess.

[B2] Rowlands I, Nicolas E, Huntingdon P (2004). Scholarly communication in the digital environment: what do authors want?.

[B3] Public Library of Science. http://www.plos.org/.

[B4] BioMed Central: the open access publisher. http://www.biomedcentral.com/.

[B5] Swan A, Brown S (2004). JISC/OSI journal authors survey report.

[B6] North Carolina State University, Scholarly Communication Center. http://www.lib.ncsu.edu/scc/index.html.

[B7] Antelman K (2004). Do open-access articles have a greater research impact?. College & Research Libraries.

[B8] Eysenbach G (2006). Citation advantage of open access articles. PLoS Biology.

[B9] Harnad S, Brody T (2004). Comparing the impact of open access (OA) vs. non-OA articles in the same journals. D-Lib Magazine.

[B10] Nicholas D, Rowlands I (2005). Open access publishing: the evidence from the authors. Journal of Academic Librarianship.

[B11] Bailey C Key open access concepts, excerpt from open access bibliography: liberating scholarly literature with E-prints and open access journals. http://www.escholarlypub.com/oab/keyoaconcepts.htm.

[B12] Anderson R (2004). Author disincentives and open access. Serials Review.

[B13] Swan A, Brown S (2004). Authors and open access publishing.

[B14] Hardie G (2004). An open access author speaks. The Scientist.

[B15] Hayden M, Suhail A (2004). Open access authors express enthusiastic support and serious reservation. The Scientist.

[B16] Weitzman J (2004). Open access and creative common sense. The Scientist.

[B17] Lawrence S (2001). Free online availability substantially increases a paper's impact. Nature.

[B18] Swan A (1999). What authors want: the ALPSP research study on the motivations and concerns of contributors to learned journals.

[B19] Doyle H, Gass A, Kennison R (2004). Who pays for open access?. PLoS Biol.

[B20] Peet R Crises and opportunities: a scientist's view ofscholarly communication. Scholarly communications in a digital world: a convocation Chapel Hill, NC, January 27–28, 2005.

[B21] Scholarly communications in a digital world: a convocation. http://www.unc.edu/scholcomdig/whitepapers/index.html.

[B22] Gasaway L Copyright issues and scholarly communications. Scholarly communications in a digital world: a convocation Chapel Hill, NC, January 27–28, 2005.

[B23] PubMed. http://www.pubmed.gov.

[B24] PubMed Central. http://www.pubmedcentral.gov/.

[B25] Rowlands I, Nicholas D (2005). Scholarly communication in the digital environment: the 2005 survey of journal author behaviour and attitudes. Aslib Proceedings.

[B26] Parthasarathy H (2005). Measures of impact. PLoS Biology.

[B27] PLoS ONE. http://www.plosone.org/.

[B28] UNC-Chapel Hill Office of Sponsored Research, annual report FY 2005. http://research.unc.edu/osr/reporting/documents/fy05appendix1.pdf.

[B29] Duke University Financial Services, financial statements 2004/2005. http://www.finsvc.duke.edu/Resources/reports/financial_reports05.pdf.

